# Nanoporous and nonporous conjugated donor–acceptor polymer semiconductors for photocatalytic hydrogen production

**DOI:** 10.3762/bjnano.12.50

**Published:** 2021-06-30

**Authors:** Zhao-Qi Sheng, Yu-Qin Xing, Yan Chen, Guang Zhang, Shi-Yong Liu, Long Chen

**Affiliations:** 1 College of Materials, Metallurgical and Chemistry, Jiangxi University of Science and Technology, Ganzhou, 341000, China,; 2 Department of Chemistry, Tianjin University, Tianjin 300072, China

**Keywords:** π-conjugated polymeric photocatalysts, donor–acceptor junctions, nanostructure semiconductors, photocatalytic hydrogen production

## Abstract

Conjugated polymers (CPs) as photocatalysts have evoked substantial interest. Their geometries and physical (e.g., chemical and thermal stability and solubility), optical (e.g., light absorption range), and electronic properties (e.g., charge carrier mobility, redox potential, and exciton binding energy) can be easily tuned via structural design. In addition, they are of light weight (i.e., mainly composed of C, N, O, and S). To improve the photocatalytic performance of CPs and better understand the catalytic mechanisms, many strategies with respect to material design have been proposed. These include tuning the bandgap, enlarging the surface area, enabling more efficient separation of electron–hole pairs, and enhancing the charge carrier mobility. In particular, donor–acceptor (D–A) polymers were demonstrated as a promising platform to develop high-performance photocatalysts due to their easily tunable bandgaps, high charge carrier mobility, and efficient intramolecular charge transfer. In this minireview, recent advances of D–A polymers in photocatalytic hydrogen evolution are summarized with a particular focus on modulating the optical and electronic properties of CPs by varying the acceptor units. The challenges and prospects associated with D–A polymer-based photocatalysts are described as well.

## Introduction

To date, fossil fuels still are the predominant energy source around the world. This leads to severe environmental problems, such as the ever-worsening global warming due to the excessive emission of greenhouse gases and the pollution by poisonous gases and particles generated during the incomplete combustions of fossil fuels. In addition, fossil fuels are limited and will be depleted. Regarding clean and sustainable energy resources, in particular solar energy has become a candidate to eventually replace fossil fuels. Among the various strategies, hydrogen production by photocatalytic water splitting is emerging as a promising approach and hot research topic. Hydrogen is regarded as a clean and recyclable energy source, which can be obtained from water and, in turn, generates water as the only product after consumption [1–3]. Inspired by natural photosynthetic systems that can convert solar energy into chemical fuels, Fujishima and Honda [4] reported the first example of hydrogen production by photocatalytic water splitting in 1972, using TiO_2_ as the photocatalyst under ultraviolet-light irradiation. Since then, numerous semiconductors have been explored for photocatalytic hydrogen production (PHP) by water splitting, which are primarily inorganic materials, such as metal oxides and sulfides [5].

Inorganic photocatalysts, however, have some inherent drawbacks. Harsh synthetic conditions, such as high pressure and temperature, are required [5]. Moreover, many reported inorganic photocatalysts contain heavy metal elements, for example, La, Bi, and Ta, which are often rare, toxic, and expensive [6]. Also, expensive noble metal-based cocatalysts (e.g., Pt) are required to improve the photocatalytic performance. As such, an ideal photocatalyst for water splitting reaction should fit the following criteria: suitable bandgap energy, high stability, wide light-absorption range, and sufficient catalytically active sites [7].

Conjugated polymers (CPs) are one of the most promising alternatives to the traditional inorganic photocatalysts. Their geometries and physical (e.g., chemical and thermal stability and solubility), optical (e.g., light absorption range), and electronic properties (e.g., charge carrier mobility, redox potential and exciton binding energy) can be easily tuned via structural design. In addition, they are of light weight (i.e., mainly composed of C, N, O, and S) [8–10]. In 1985, the first CP-based photocatalyst (i.e., poly(*p*-phenylene)) for PHP was reported, but did not attract much attention due to the low hydrogen evolution rate (HER) [11]. In 2009, Wang et al. reported a novel metal-free polymeric photocatalyst (i.e., graphitic carbon nitride (g-C_3_N_4_)), which could efficiently reduce protons to generate hydrogen under visible-light irradiation, for the first time. After that, g-C_3_N_4_ triggered substantial research interest [12–14]. Various strategies have been developed to improve the PHP activity of g-C_3_N_4_, such as introducing heterojunctions [15–17], copolymerization [18–20], doping with other elements [21–23], and the control of end groups [24]. Meanwhile, various types of CPs have been applied for PHP, including conjugated porous polymers (CPPs) [25–27], linear conjugated polymers (LCPs) [28–30], conjugated triazine frameworks (CTFs) [31–33], and covalent organic frameworks (COFs) [34]. Notably, a record HER of up to 307 mmol·h^−1^·g^−1^ has been achieved with a pyrene–bithiophene-based porous polymer as the photocatalyst [35].

During PHP by water splitting, first, the photocatalyst is excited by photons, and photoexcited electrons hop into the lowest unoccupied molecular orbital (LUMO) or conduction band (CB), while holes remain in the highest occupied molecular orbital (HOMO) or valence band (VB). Second, the electron–hole pairs are transferred to the surface through thermodynamic driving forces and are captured by H^+^ and a sacrificial electron donor (SED) in water, which eventually produces H_2_ [36–37]. Accordingly, three important factors, that is, light-harvesting ability, mobility of the photogenerated charge carriers, and electron–hole separation efficiency, need to be considered simultaneously to design efficient photocatalysts.

**Figure 1:**
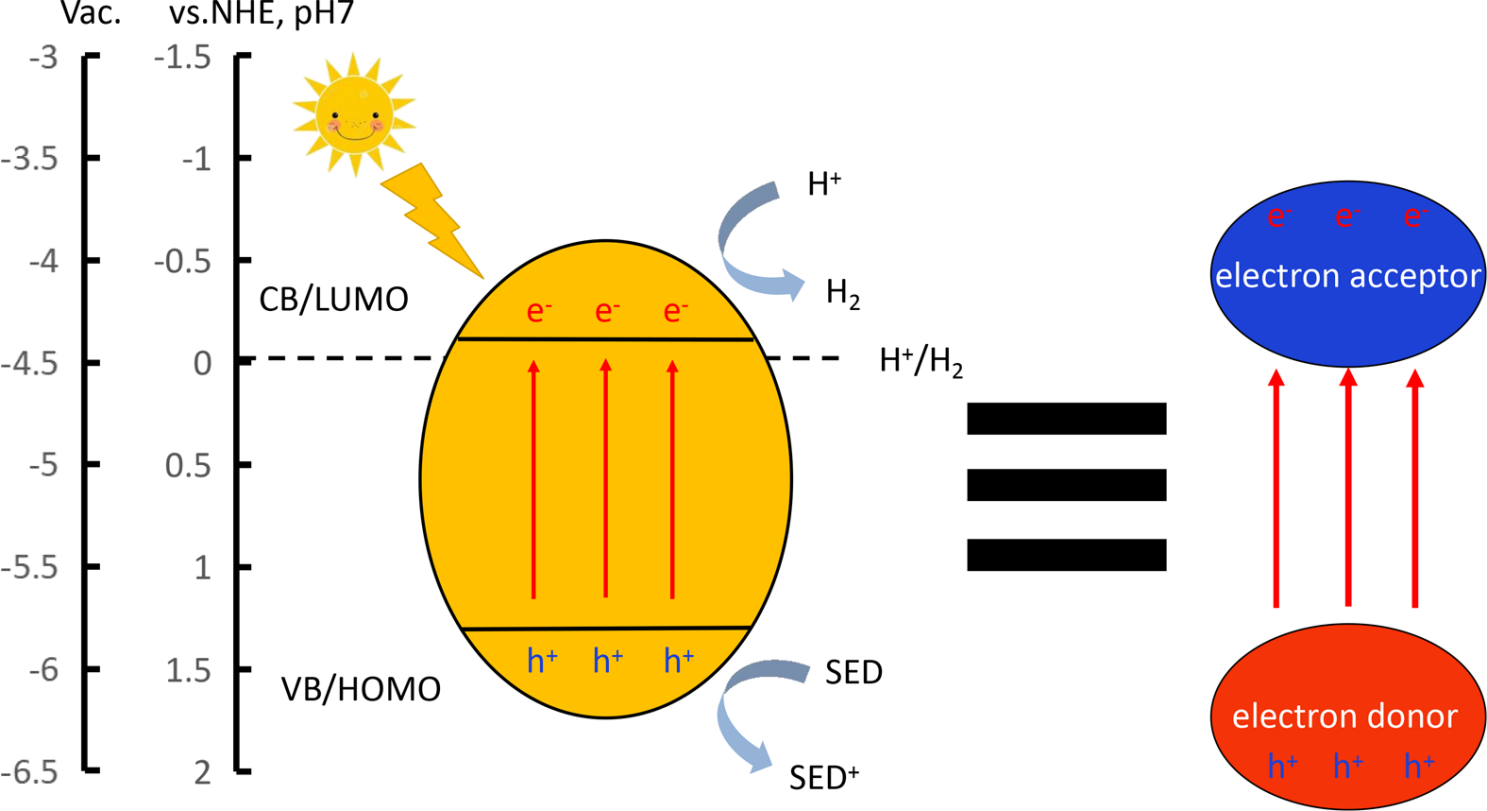
(Left) Schematic diagram of the mechanism of semiconducting catalyst-mediated photocatalytic hydrogen production (CB: conduction band, VB: valence band, SED: sacrificial electron donors). (Right) Charge separation in a CP-based semiconductor via photoinduced D→A charge transfer.

Organic photocatalysts with narrow bandgap and high charge carrier mobility could, therefore, facilitate light harvesting and the reduction of protons [38]. In terms of structural design, D–A polymers are a good platform to narrow the bandgap, enhance the charge carrier mobility and promote electron–hole separation (Figure 1) by selectively tuning the donor and acceptor parts within the conjugated backbones [39–40]. The D–A architecture has been widely employed in high-performance organic optoelectronic devices, such as organic photovoltaics, organic field-effect transistors, nonlinear optics, and organic light-emitting diodes (OLEDs) [41]. However, it was only in recent years that researchers have started to use D–A design strategy to develop CP-based photocatalysts for PHP. As there is growing interests in this field, a timely review article about this area would be quite instructive and necessary [42].

This minireview article summarizes D–A-type conjugated polymers as photocatalysts for PHP, including LCPs, CPPs, CTFs, and COFs with a particular focus on modulating the electron-accepting segments, that is, triazine, pyridine, benzothiadiazole, dibenzothiophene-*S*,*S*-dioxide, and cyano moieties. By tailoring the functional groups and geometries of the polymer framework, the influence of different factors on the photocatalytic activity can be systematically investigated and, consequently, the structure–performance relationships are unveiled. Herein, the donor and acceptor fragments in the polymer structures are highlighted in red and blue, respectively.

## Review

### Donor–acceptor conjugated polymers

#### Triazine-based conjugated polymers

**Figure 2:**
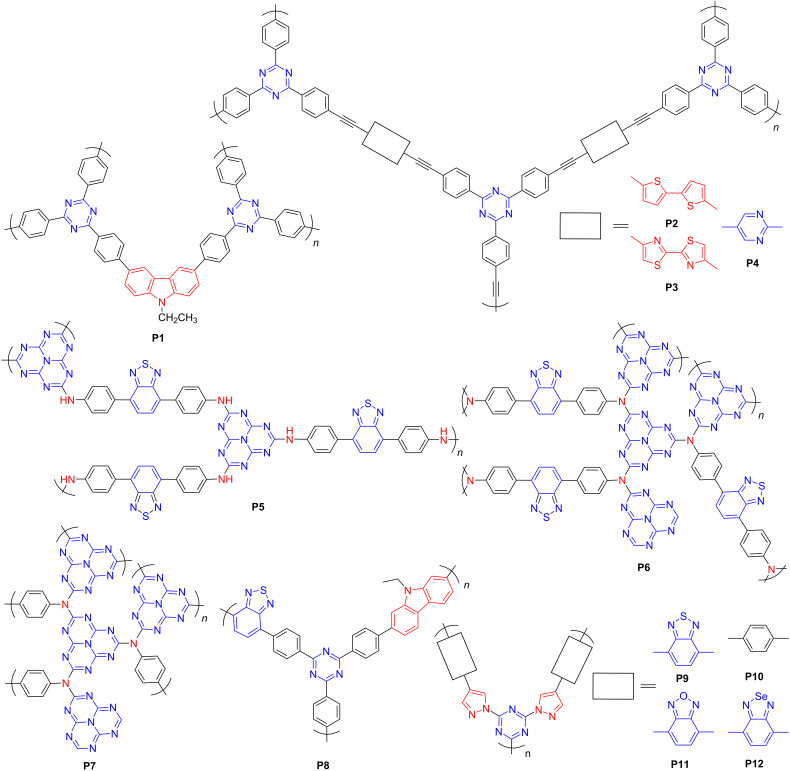
Structures of triazine-based conjugated polymers.

*s*-Triazine and tri-*s*-triazine (heptazine), as the building blocks of carbon nitride, represent two of the most widely studied acceptor units [43–44]. In contrast to the conventional trimerization of nitrile under harsh conditions [43], Tan et al. developed a new condensation reaction of aldehydes with amidines to construct covalent triazene frameworks (CTFs) under mild conditions [45]. They investigated the effects of donor units with different heteroatoms on the photocatalytic performance of CTFs. The carbazole–triazine-containing CTF **P1** (Figure 2) triggered a strong intramolecular charge transfer (ICT) and achieved a high HER of 538 μmol·h^−1^ (50 mg).

Bojdys and co-workers reported a series of CTFs with diverse functional moieties (e.g., heterocycles containing S and/or N) at the edges of the frameworks to delicately tune the bandgaps of conjugated polymers. Among them, three polymers, that is, **P2**, **P3**, and **P4** (Figure 2), showed suitable bandgaps of ca. 2.2 eV, and conferred HERs of 4.72, 9.15, and 2.90 μmol·h^−1^ (10 mg), respectively, under visible-light irradiation [46–47]. The bandgaps of the CTFs can be easily tuned to optimize the photocatalytic activity by introducing different functional groups. Besides using a single kind of electron acceptor, researchers also attempted to incorporate multiple electron acceptors to construct CPs for PHP. For instance, Thomas et al. [48] prepared several porous polymers with benzothiadiazole and heptazine moieties as the acceptors and an aminobenzene segment as the donor. By adjusting the molar ratio between the monomers, secondary (**P5**, benzothiadiazole/heptazine = 2:3) or tertiary amine-linked polymers (**P6**, benzothiadiazole/heptazine = 4:3) (Figure 2) were obtained. **P5** exhibited a smaller bandgap of 1.99 eV. Moreover, the photoluminescence spectra indicated an improved separation efficiency of photogenerated charges of **P5**. A stable H_2_ evolution rate of 32 μmol·h^−1^ (20 mg) was achieved for HMP-3_2:3, which was several times higher than those of the triazine-amine-based **P7** (Figure 2) and bulk g-C_3_N_4_. Hence, incorporation of more than one kind of electron acceptors in the polymer structure could enhance the photocatalytic efficiency.

**Figure 3:**
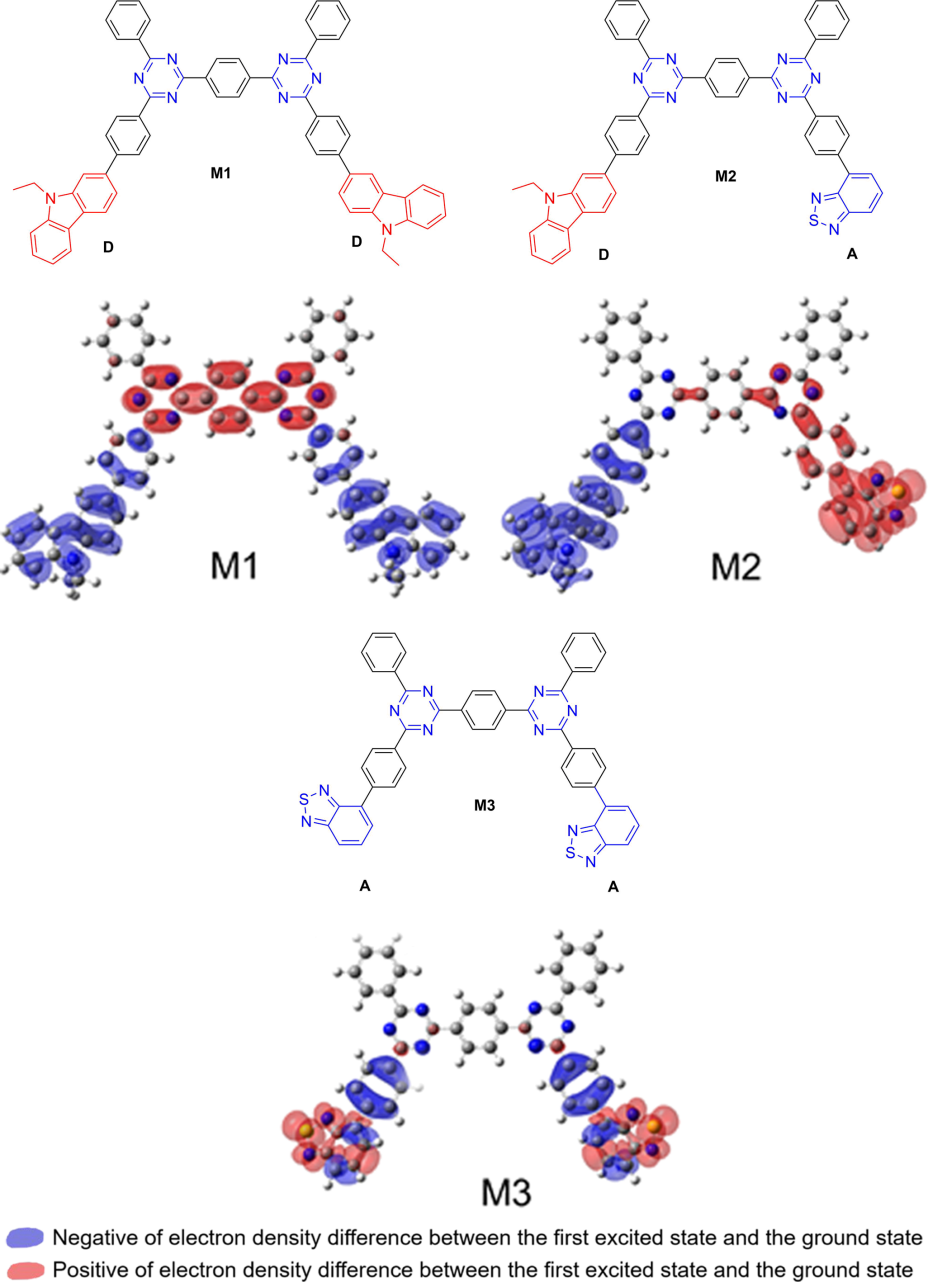
Proposed model fragments and electron density differences of **M1**–**M3** in **P8**. Adapted with permission from [49]. Copyright (2019) American Chemical Society. This content is not subject to CC BY 4.0.

In addition, Tan et al. [49] developed several D–A CPs with carbazole (D), benzothiadiazole (A1) and triazine (A2) fragments. The photocatalytic activities of CPs with different D/A1 ratios were systematically investigated and compared. For example, **P8** (D/A1 = 3:7) (Figure 2) yielded the highest HER (966 μmol·h^−1^, 50 mg), which was consistent with the highest photocurrent response, smallest bandgap, and suppressed recombination of charge carriers in **P8**. The electron density distributions of three kinds of triazine-based fragments containing carbazole (**M1**), or both carbazole and benzothiadiazole (**M2**), and benzothiadiazole (**M3**) (Figure 3) were further calculated to support these results. **M1** and **M2** showed better charge separation than **M3**. In particular, **M2** exhibited superior charge separation when considering spatial effects among the three fragments. Consequently, increasing the fraction of **M2** in the polymers could be beneficial to the photocatalytic efficiency.

He et al. developed two pyrazole-triazine-based CTFs, that is, **P9** (A–D–A) and **P10** (D–A) (Figure 2) by a metal-free catalyzed approach [50]. Compared with **P10**, introducing a benzothiadiazole unit into **P9** effectively reduced the optical bandgap from 2.94 eV to the ideal value of 2.33 eV. They further probed the influence of substituting the S atom in the benzothiadiazole group with O or Se atoms on the optoelectronic and photocatalytic properties of the CTFs **P11** and **P12** (Figure 2) [51]. Introducing an O atom boosted the ICT and electron–hole separation due to the higher electron negativity; but it lowered the LUMO level, which as a result, suppressed the PHP reaction. Incorporating a Se atom led to a redshifted light absorption, but decreased the charge carrier mobility due to the reduced aromaticity of the architecture. Consequently, the HERs declined in both cases. These results reveal that slight structural changes could significantly modulate the properties and photocatalytic activities of CTFs.

#### Pyridine-based conjugated polymers

Pyridine, as a nitrogen-containing benzene analogue, was incorporated into linear conjugated polymers as early as in the 1990s. The CPs exhibited distinctive photocatalytic activity for H_2_ production under visible light [52]. With the extensive studies of nitrogen-rich triazine- and heptazine-based photocatalysts for hydrogen production, pyridine and its derivatives as nitrogen-containing acceptors have attracted great interest again, and D–A architectures for photocatalytic water splitting were synthesized.

**Figure 4:**
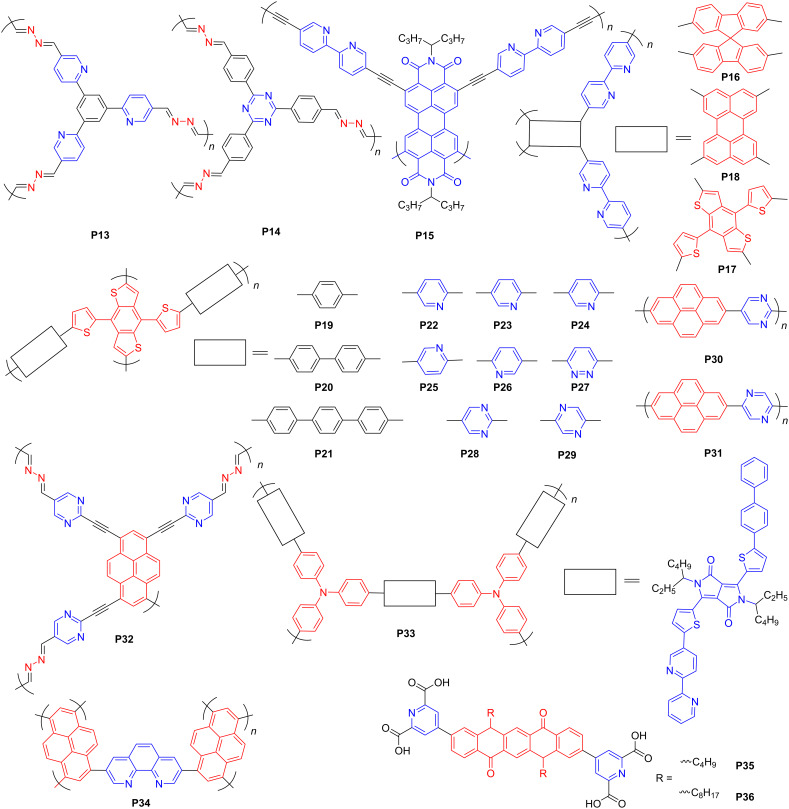
Schematic representation of structures of pyridine-contained conjugated polymers.

In 2015, Lotsch et al. [53] reported four COFs with benzene, pyridine, pyrimidine, and triazine as the core to disclose the effects of nitrogen atoms on the photocatalytic activities of these COFs. It showed a linear correlation between HER and the content of nitrogen atoms in the COF skeleton. However, HER is affected by the interplay of several factors, including surface area, light absorption range, crystallinity, charge transfer, and separation. Two azine-linked COFs with three pyridine segments neighboring the central benzene or triazine fragment were prepared to make comparisons, that is, the 1,3,5-tri(pyridin-2-yl)benzene based COF **P13** and the benzene-triazine-based **P14** (Figure 4). Both of them showed a similar AA eclipsed stacking in the crystal structures, but the lower symmetry of **P13** resulted in worse crystallinity. Particularly, the D–A interactions between the pyridine and the benzene rings gave rise to disorder and reduced crystallinity even further. As a result, the HER of **P13** (0.42 μmol·h^−1^, 5 mg) was one order of magnitude lower than that of **P14** (8.52 μmol·h^−1^, 5 mg) [53].

Cai et al. [54] reported a series of perylene diimide (PDI)-based n-type porous CPs (Figure 4). The HERs of the polymers with phenyl or biphenyl moieties as donors were lower than that of the pure acceptor polymer **P15** (7.2 μmol·h^−1^, 3.5 mg). Although the benzene group could serve as a weak donor in some cases, it is difficult to render effective D–A interactions. For instance, Yu and co-workers [55] combined electron-deficient PDI or electron-rich chromophores (including spirofluorene, perylene, and 4,8-di(thiophen-2-yl)benzo[1,2-*b*:4,5-*b*']dithiophene (DTBDT) with bipyridine moieties to form several porous polymers (**P15**–**P18**) (Figure 4) to investigate the relationship between D–A interactions and photocatalytic properties. PDI-containing polymers, such as **P15,** normally confer smaller HERs than the others. **P18**, composed of strong electron-donating and bipyridine segments, rendered the best HER, which was attributed to its enhanced light absorption, better wettability, and more efficient charge separation. Based on DTBDT monomer, many porous polymers (**P19**–**P29**) (Figure 4) were further prepared to study the effects of different acceptors on the photocatalytic performance during water splitting [56]. PCPs incorporating nitrogen-containing heterocycles showed a better photocatalytic performance than those with oligophenylene moieties, which is due to the favorable internal polarization and coordinating sites rendered by nitrogen-containing heterocycles. Among them, the DTBDT–pyrazine-based **P29** achieved the best HER of 106.9 μmol·h^−1^ (12 mg) due to the suitable N substitution.

Pyrene as a well-known chromophore has been widely used in OLEDs and fluorescent probes due to its high photoluminescent quantum yield. Jiang et al. [57] introduced various sorts of nitrogen-containing fragments in pyrene-based polymers to investigate their photocatalytic performance, for example, **P30** and **P31** (Figure 4). Interestingly, different from the above examples, the pyrimidine-containing **P30** (18.7 μmol·h^−1^, 50 mg) exhibited a much higher HER than **P31** with pyrazine moieties (8.6 μmol·h^−1^, 50 mg). Lotsch et al. [58] also developed several pyrene-based COFs with peripheral nitrogen-containing or nitrogen-free aromatic units, among which the pyrene–pyrimidine-based **P32** (Figure 4) with the lowest nitrogen content showed the highest HER. Nitrogen heterocycles within polymers could optimize wettability, bandgap, charge transport, and separation.

Motivated by the planar configuration and strong electron-acceptor capability of diketopyrrolopyrrole (DPP) [59–61], Li and co-workers [62] combined DPP with triphenylamine and bipyridine groups to synthesize D–A polymers. A HER of 9.73 μmol·h^−1^ (3.5 mg) was achieved for **P33** (Figure 4) due to the wide spectral range of the photoresponse. The apparent quantum yield (AQY), as a vital experimental parameter for photocatalytic performance, exceeded 9% at 420 nm, which is, to date, the highest among all reported conjugated polymer-based photocatalysts. In addition, phenanthroline–pyrene-based D–A polymers were studied [63], with polymer **P34** (phenanthroline/pyrene = 1/3) (Figure 4) exhibiting the highest HER of 42 μmol·h^−1^ (10 mg). Additionally, Hua et al. [64] constructed two D–A supramolecular architectures with different alkane chains, that is, **P35** and **P36** with n-butyl and n-octyl chains, respectively (Figure 4), by self-assembling quinacridone and pyridine-2,6-dicarboxylic acid. It was revealed that the longer alkyl chain was harmful to the D–A interactions in CPs. Thus, nitrogen-containing aromatic segments can effectively mediate the internal polarization of the polymeric framework and thus enhance the PHP performance.

#### Benzothiadiazole-based conjugated polymers

The benzothiadiazole (BT) moiety as a strong electron acceptor features high planarity and good wettability due to incorporating N and S atoms. In 2016, Wang and co-workers [65] investigated the properties of a linear conjugated polymer and a 3D counterpart by varying the substitution position on the benzene ring. Interestingly, the linear polymer exhibited more effective D–A interactions than the 3D counterpart due to the enhanced conjugation of the former with a planar architecture.

**Figure 5:**
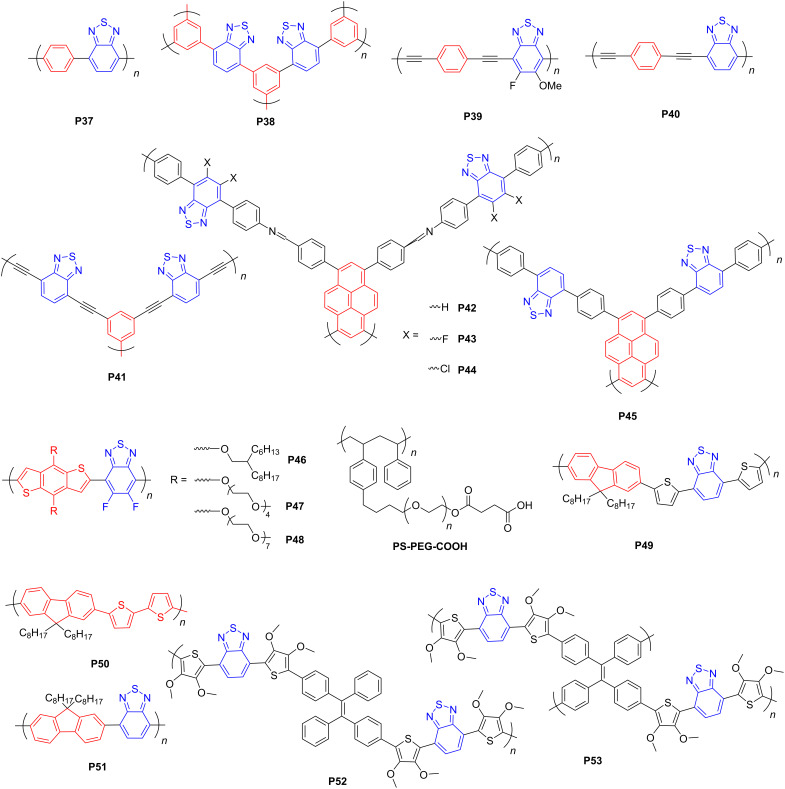
Schematic representation of structures of benzothiadiazole-based conjugated polymers.

**Figure 6:**
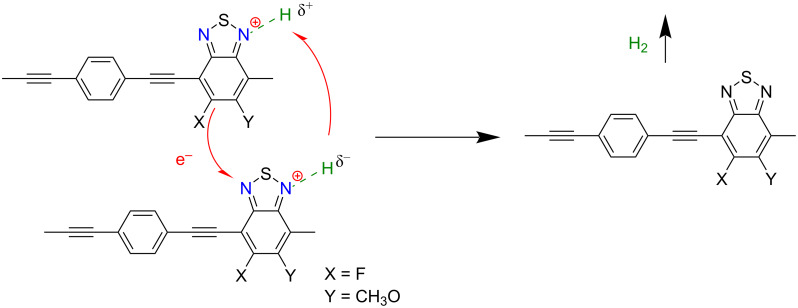
The mechanism of PCET-enhanced H_2_ formation; see [66].

Two ethynyl-linked benzene–benzothiadiazole-based porous polymers, that is, **P39** and **P40** (Figure 5) were synthesized to investigate the influence of fluorine substitution at BT on the charge carrier mobility and catalytic activity [66]. The mechanism of proton-coupled electron transfer (PCET) (Figure 6) was offered. It suggests for both linear and 3D polymers that single meta-F substitution renders a higher photocatalytic activity than double F substitution, and the polymer with the methoxy group at the meta site and the F atom at the para site yields the highest HER due to the excellent mobility of photogenerated charge carriers and the broad light absorption range. DFT calculations also verified that incorporating F on the para carbon atom of the BT unit strengthens the intermolecular interactions and thus enhances the charge transfer effect among the polymer chains. This is due to the interaction between two p orbitals from the meta carbon atom on one molecule and a N atom on the other. In addition, a methoxy group at the meta position intensified the intermolecular interaction, which, however, was weakened by F substitution at the meta position. Consequently, the linear **P39** (Figure 5) with both methoxy group and F on BT afforded the highest HER of 399 μmol·h^−1^ (30 mg). This suggests that selectively functionalizing the BT unit with F could greatly enhance the charge transfer in BT-based polymers.

**Figure 7:**
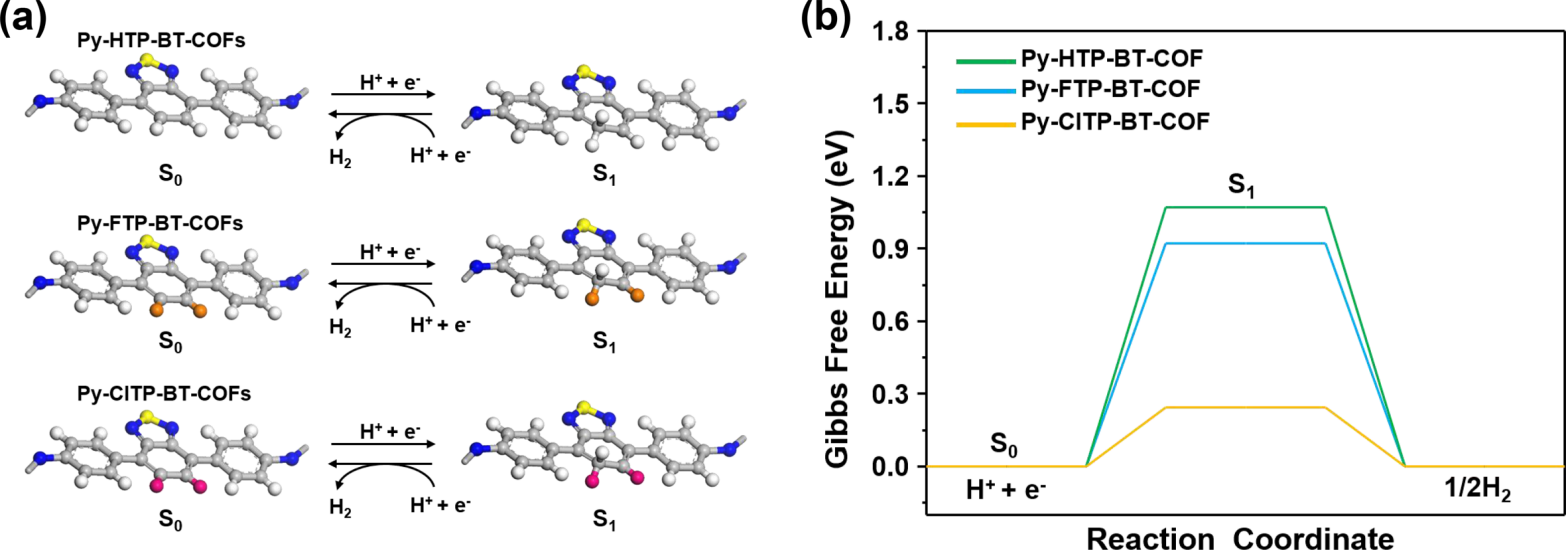
(a) Proposed reaction pathway for H_2_ evolution (single-site reaction) on the halogen-substituted carbon atom of **P42**–**P44** (grey: C; white: H; yellow: S; blue: N; orange: F; pink: Cl). (b) Free-energy diagrams for H_2_ evolution via a single-site reaction pathway at 0 V vs RHE on the halogen-substituted carbon atom of COFs. Reproduced from [67]. Copyright © 2020 Wiley-VCH GmbH. Used with permission from Chen et al., Modulating Benzothiadiazole-Based Covalent Organic Frameworks via Halogenation for Enhanced Photocatalytic Water Splitting, Angew. Chem. Int. Ed., John Wiley and Sons. This content is not subject to CC BY 4.0.

Besides fluorine substitution, several halogenated BT-based imine-linked COFs were reported by Chen et al. [67] to investigate the effects of halogen atoms on the photocatalytic performance. The Cl-substituted COF with pyrene and BT exhibited the best HER (177.50 μmol·h^−1^, 20 mg) due to low charge recombination and strong photoinduced charge transfer. Furthermore, DFT calculations (Figure 7) indicated that incorporating halogen atoms in both **P43** and **P44** (Figure 5) reduces the energy barrier for forming H* intermediates on polymer surface. Consequently, halogen substitution on the polymer is an approach to evoke enhanced charge separation and improve the HER of CPs.

In 2018, Jiang et al. [68] systematically studied the effects of π-linking groups, that is, benzene or biphenyl moieties in pyrene–benzothiadiazole-based polymers, for example, **P45** (Figure 5). The D–π–A-type compound **P45** possesses an extended π-conjugation along the backbone and consequently has an enlarged visible-light absorption and enhanced electronic conductivity compared with π-linker-free pyrene–benzothiadiazole-based polymers. After deposition of Pt as the cocatalyst, **P45** exhibited the most prominent photocatalytic activity for H_2_ evolution (29.6 μmol·h^−1^, 100 mg) under visible light. These results suggest that the D–π–A architecture might be better than the conventional D–A counterpart for designing high-performance polymer-based photocatalysts for H_2_ evolution.

Although the BT group can increase the wettability of polymers [50], most BT-based polymers are difficult to disperse in water. To overcome the issue of poor dispersibility of BT-based polymers, Huang et al. [69] synthesized two BT-based linear CPs with oxygen-containing branched side chains. Different from long alkyl chains, the polymers incorporating the oligo(ethylene glycol) side chains (**P48**) (Figure 5) showed considerably improved charge separation and transport and could be dispersed in water without any co-solvent. Similar to the N atoms in BT units, oxygen atoms can also adsorb H^+^ in water to form the polymer/water interface.

Tian et al. [70] prepared several BT-based polymer dots (Pdots) with functionalized polystyrene (PS-PEG-COOH) (Figure 5) to improve the wettability of polymers. In contrast to the pristine BT-based polymers (**P49**–**P51**) (Figure 5), the Pdots were dispersed in water. The Pdots of **P49** rendered one of the highest HER (150 μmol·h^−1^, 3 mg) among all Pdots, which was attributed to the water solubility, broad light absorption and abundant catalytic sites of BT-based Pdots. In addition, Hu et al. [71] synthesized hyper-branched Pdots by co-assembling pristine polymers (**P52** and **P53**) and hydrophilic PEG_45_-*b*-PMMA_103_ (Figure 5). The hyperbranched Pdots yielded higher HERs (as high as 16.8 μmol·h^−1^ (20 mg) for **P53**) than the linear Pdot **P52**, due to the shortened charge transport pathway, 3D transport, and more negative reduction potentials of the hyperbranched Pdots. Araujo et al. [72] studied several BT-containing small molecules with A–D–A architectures through theoretical calculations. They found that small molecules could also mediate photocatalytic water splitting into hydrogen and oxygen.

#### Dibenzothiophene-*S*,*S*-dioxide-based conjugated polymers

**Figure 8:**
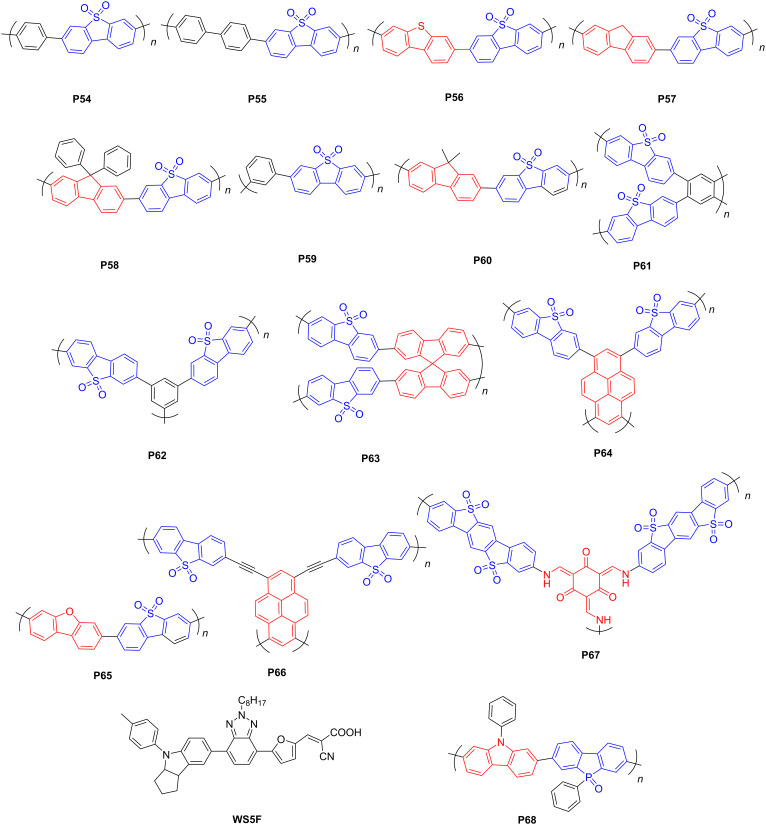
Schematic representation of structures of dibenzothiophene-*S*,*S*-dioxide-based CPs.

Recently, dibenzothiophene-*S*,*S*-dioxide (FSO) has been extensively applied to construct D–A CPs due to its planar architecture, matched energy levels, and strong electron withdrawal property. The sulfonyl groups could not only increase the wettability through the O^…^H hydrogen bonding, but also function as the sites for photocatalytic proton reduction in water [73]. Cooper and co-workers [74] first reported FSO-based CPs as catalysts for PHP in 2016. The linear FSO–phenyl-based polymer **P54** (Figure 8) yielded a moderate HER of 92 μmol·h^−1^ (25 mg) under visible light.

Subsequently, Wang et al. [75] combined FSO with biphenyl, dibenzothiophene, or fluorene segments to construct D–A CPs (**P55**–**P57**). Experimental studies showed that the dibenzothiophene-containig polymer **P56** (Figure 8) exhibited an outstanding AQY of 6.8% at 420 nm. Both dibenzothiophene and fluorene moieties possess planar structures compared with biphenyl group, yet dibenzothiophene has a more extended conjugation than fluorene due to the existence of a saturated 9-C atom in fluorene. Therefore, **P56** with the most extended conjugation facilitated more efficient charge transfer and separation. High planarity and extended conjugation of a polymer skeleton are crucial to realize efficient photocatalytic water splitting. Liu et al. [76] integrated FSO with fluorene or 9,9-diphenylfluorene segments to prepare two D–A polymers (**P57** and **P58**) (Figure 8). The photocatalytic activities of these D–A polymers increased with enhancing the planarity of the polymer backbones, due to improved charge transfer and increased light absorption in more planar conjugated systems. Therefore, the fluorene-containing polymer **P57** (Figure 8) exhibited the best performance among the three D–A polymers with a HER of 50.4 μmol·h^−1^ (10 mg).

Comparing the photocatalytic performance of linear CPs and their 3D counterparts, many research articles claimed that linear CPs render higher HERs than porous CPs due to the superior charge carrier mobility of linear polymers [65–66,71]. However, Cooper et al. [77] reasoned that porous polymers might be better than linear counterparts to promote photocatalytic hydrogen evolution as long as the charge carrier transport was not compromised by the 3D structures. This claim was exemplified by the 9,9'-spirobifluorene–FSO-based porous polymer **P63** (Figure 8), which exhibited the best catalytic performance under visible light among the linear and porous polymers in that work, with a HER of 77.65 μmol·h^−1^ (25 mg). Jiang et al. [78] reported two pyrene–FSO-based porous CPs with different linking positions with respect to dibenzothiophene-*S*,*S*-dioxide, for example, the 3,7-position-linked polymer **P64** (Figure 8). **P64** afforded a higher HER of 284.85 μmol·h^−1^ (50 mg) under visible light without cocatalyst than the 2,8-position-linked compound due to the more extended conjugation and enhanced coplanarity of **P64**. As outlined above, judiciously selecting donor and acceptor components and designing 3D polymers could give rise to impressive performances during PHP.

Recently, Cooper and co-workers studied the effects of replacing the C9 of the fluorene moiety in polymer **P57** with heteratoms [79]. The oxygen-containing dibenzo[*b*,*d*]furan–FSO-based polymer **P65** (Figure 8) showed a higher HER (147.1 mol·h^−1^, 25 mg) than **P57** under visible light. This was attributed to the improved wettability of **P65** induced by the additional electronegative O atom and to the decreased ionization potential of trimethylamine (TEA) accelerating its oxidation through the higher driving force of **P65** for overall TEA oxidation [79]. In addition, Wang et al. [80] prepared a porous polymer with pyrene core and F atom-functionalized FSO segments (Figure 8). Different from the BT-based polymers mentioned above, incorporating F atoms at the FSO moiety resulted in a decreased HER. The authors reasoned that **P64** with the sulfonyl group could efficiently concentrate as well as output electrons and build excellent electron-output “tentacles” and therefore increase the HER. Chen et al. [81] reported an ethynyl-bridged FSO–pyrene-based polymer (**P66**) (Figure 8) to further extend the conjugation. The water contact angle measurements showed that the wettability was enhanced with increasing the content of FSO units. **P66** achieved an AQY of 8.5%.

Cooper et al. [82] systematically investigated the influence of plasma treatments on conjugated polymers as photocatalysts, for example, the fluorine–FSO-based **P60** (Figure 8). For **P60**, a short time of plasma treatment significantly enhanced the wettability and photocatalytic performance due to the plasma oxidizing the polymer. In addition, they [83] prepared a crystalline benzo[1,2-*b*:4,5-*b*']bis[1]benzothiophene sulfone-containing covalent organic framework (**P67**) (Figure 8), which exhibited a higher HER than its amorphous or semicrystalline counterparts. Dye sensitization further enhanced the photocatalytic activity of **P67**. Remarkably, incorporating a near-infrared absorbing dye (**WS5F**) into **P67** (252.5 μmol·h^−1^, 25 mg**)** further improved the HER to 497.5 mmol·h^−1^ (25 mg), which was attributed to the enhanced light absorption and photoinduced **WS5F**-to-**P67** charge transfer. Similar to the works on the FSO moiety, Chou et al. explored the phenylbenzo[*b*]phosphindole 5-oxide-containing polymer **P68** (Figure 8), which exhibited the highest AQY of 14.92% at 420 nm due to its suitable light absorption range [84].

#### Cyano-containing conjugated polymers

**Figure 9:**
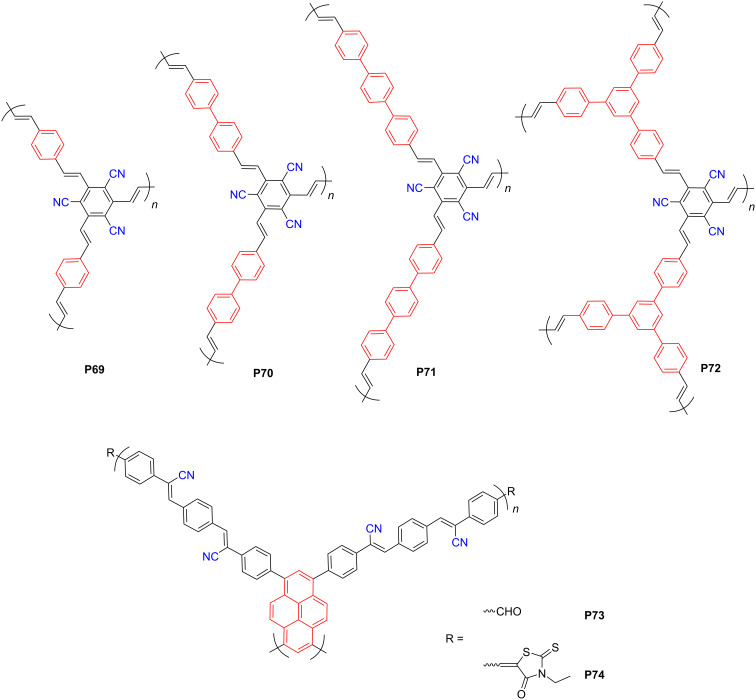
Schematic representation of structures of cyano-based CPs.

As summarized above, the D–A architecture is a fundamental design principle toward high-performance polymer-based photocatalysts, and selecting proper donor and acceptor components significantly influences the eventual photocatalytic activity. Apart from the aforementioned acceptors, the cyano moiety as a strong electron-withdrawing group, has been widely used to design organic semiconductors for diverse applications, such as organic solar cells [85], organic light-emitting diodes [86], and organic field-effect transistors [87]. Researchers have also incorporated cyano moieties into the design of D–A polymers for PHP applications. For instance, Zhuang et al. [88] developed several cyano-substituted porous polymers, which, however, only led to moderate HERs, such as **P71** (Figure 9) with a HER of 45.4 μmol·h^−1^ under visible light. Subsequently, Wang et al. reported two olefin-linked, cyano-substituted, benzene–1,3,6,8-tetrephenylpyrene-based 2D polymers (**P73** and **P74**). **P74** rendered a higher HER of 106 μmol·h^−1^ (50 mg) than **P73** (68 μmol·h^−1^, 50 mg) due to the strongly electron-withdrawing 3-ethylrhodanine fragments in the periphery [89].

### CP-based heterojunctions for PHP applications

**Figure 10:**
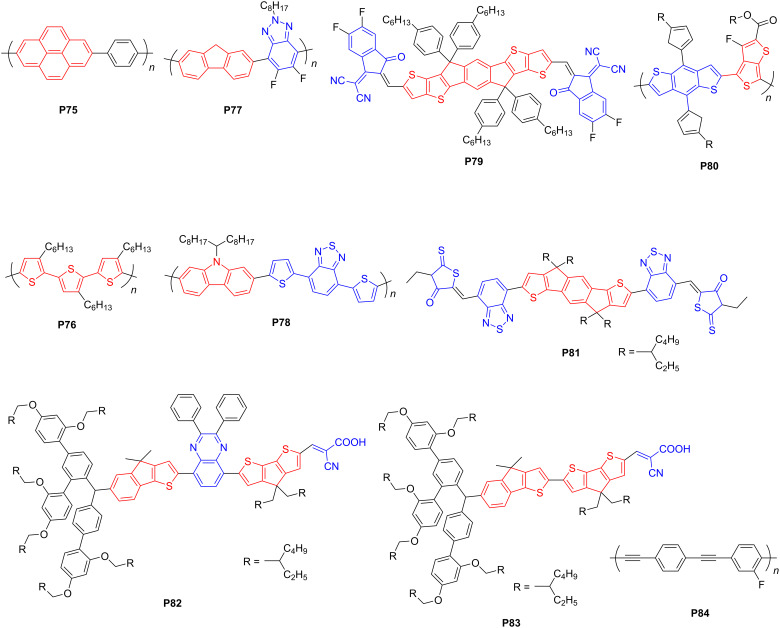
Schematic representation of structures of CPs for heterojunctions.

Besides D–A polymers, heterojunctions between CPs and other semiconductors have also been developed to facilitate the intermolecular charge transfer and transport. The interactions between the CPs and various semiconductors in the heterojunctions could involve strong covalent bonds, ionic bonds, or hydrogen bonds [90–91]. Researchers have also attempted to use heterojunctions as photocatalysts for PHP. For example, Wang et al. combined a pyrene-based polymer (**P75**) (Figure 10) with g-C_3_N_4_ to form a heterojunction [92]. The interface between **P75** and g-C_3_N_4_ restrained charge recombination and promoted proton reduction. Peng et al. also prepared a photocatalyst with a surficial heterojunction composed of **P76** (Figure 10) and g-C_3_N_4_ by facile rotary evaporation [93]. Using ascorbic acid as the sacrificial agent, the AQY of the heterojunction increased to 59.4% at 500 nm. It suggests that polymer/polymer heterojunctions could effectively improve the intermolecular charge transfer and suppress charge recombination. In addition, Tian et al. developed a series of polymer/polymer heterojunctions with CPs and g-C_3_N_4_ [94]. In particular, the heterojunction between benzotriazole–fluorene-based **P77** (Figure 10) and g-C_3_N_4_ endowed a high AQT of 33.5% at 450 nm.

Inspire by the architectures of organic solar cells, Cooper et al. selected a ITIC-based polymer as acceptor and a BT–carbazole-based one as donor to form polymer/polymer heterojunctions [95]. Due to the formation of abundant donor–acceptor interfaces, **P78**/**P79** (Figure 10) exhibited the highest HER of 191.82 μmol·h^−1^ (1.15 mg) [95]. Recently, McCulloch and co-workers discovered another D/A heterojunction (**P80**/**P81**) (Figure 10) as photocatalyst for hydrogen evolution. By stabilizing the surfactant employed during synthesis of the heterojunction, ITIC-containing **P80** as the acceptor could intimately blend with the donor **P81**, which rendered HERs up to 128.85 μmol·h^−1^ (2 mg) [96].

Regarding heterojunctions with TiO_2_, for instance, Hua et al. used two indeno[1,2-*b*]thiophene-based organic dyes (**P82** and **P83**) (Figure 10) to sensitize TiO_2_ to harvest near-infrared light [97]. Consequently, the average HERs of **P82**/TiO_2_ and **P83**/TiO_2_ with Pt as cocatalyst dramatically increased to 11.3 and 3.9 times, respectively, as high as that of using TiO_2_ only. Chen et al. attempted to incorporate several CPs, that is, **P37**, **P40,** and **P84** (Figure 5 and Figure 10), with TiO_2_ and formed binary composites [98–100]. Compared with pristine CPs as photocatalysts, the photocatalytic activities of the heterojunctions increased by around 18, 7, and 2 times, respectively. Subsequently, the same research group developed a **P40** flake/CdS heterojunction with broad visible light absorption (400–700 nm) and high photogenerated charge separation rate [101]. This heterojunction realized an AQY of up to 7.5% at 420 nm. By in situ solvothermal growth of CdS nanoparticles on ultrathin polyimide (PI) nanosheets, Zou et al. [102] achieved a HER of 30.65 μmol h^−1^ (50 mg) with 15% CdS/PI as the photocatalyst, which was nearly 5 and 60 times as high as the performance of pristine CdS and 1% Pt/PI, respectively. This suggests that organic/inorganic heterojunctions show higher photocatalytic performance than the single components of CPs.

Table 1 summarizes the main optoelectronic properties and HERs of the CP-based photocatalysts introduced in this review article.

**Table 1:** Summary of the bandgaps, cocatalysts, and properties related to H_2_ evolution of quintessential CP photocatalysts.

CPs	Bandgap (eV)	LUMO/HOMO (eV)	Cocatalyst	SED	HER (μmol·h^−1^)	HER (μmol·g^−1^·h^−1^)	AQY (%)	Ref.

**P1**	2.17	−0.86/1.31	Pt	TEOA	538^a^	10760^a^	4.07	[45]
**P3**	2.21	—	Pt	TEOA	ca. 2.90^b^	ca. 290^b^	—	[46]
**P4**	2.16	—	Pt	TEOA	9.15^b^	915±10^b^	—	[47]
**P5**	1.99	—	Pt	TEOA	32^b^	1600^b^	—	[48]
**P8**	2.11	−0.76/1.35	Pt	TEOA	966^a^	19320^a^	22.8	[49]
**P9**	2.33	−0.42/1.91	Pt	TEOA	50^a^	1000^a^	3.6	[50]
**P11**	2.37	−0.44/1.93	Pt	TEOA	22^a^	440^a^	1.43	[51]
**P13**	2.1	—	Pt	TEOA	0.42^c^	83.83^c^	4.15	[53]
**P15**	2.4	−0.90/1.5	—	TEA	7.2^c^	2057^c^	—	[54]
**P18**	2.45	—	Pt	TEA	6.5^a^	1857^a^	0.34	[55]
**P29**	ca. 1.9	ca. −1.88/0.7	—	TEA	106.9^c^	8908.3^c^	—	[56]
**P30**	2.53	−1.00/1.33	Pt	TEOA	18.7^d^	374^d^	1.1	[57]
**P32**	1.94	−0.8/1.14	Pt	TEOA	0.98^c^	98^c^	—	[58]
**P33**	2.32	−1.3/0.9	—	TEOA	9.73^a^	2780^a^	9.60	[62]
**P34**	1.85	−0.39/1.46	Pt	TEOA	42^b^	4200^b^	1.5	[63]
**P35**	1.76	−0.96/0.8	—	AA	19.68^b^	656^b^	—	[64]
**P37**	2.17	−0.89/1.28	Pt	TEOA	116^a^	2320^a^	4.01	[65]
**P39**	2.12	−0.55/1.57	—	TEOA	399^a^	13300^a^	5.7	[66]
**P44**	2.36	−1.58/0.78	Pt	AA	177.50^a^	8875^a^	8.45	[67]
**P45**	2.21	−1.00/1.22	Pt	TEOA	29.6^a^	296^a^	—	[68]
**P48**	1.69	−0.76/0.93	—	AA	32^a^	12800^a^	0.3	[69]
**P49**	1.98	−0.90/1.08	—	AA	150^a^	50000^a^	0.6	[70]
**P53**	1.97	−0.82/1.15	—	AA	16.8^a^	840^a^	0.9	[71]
**P54**	2.70	—	—	TEOA	92^a^	3680^a^	—	[74]
**P56**	3.64	−1.77/1.87	—	TEOA	170^a^	3400^a^	6.8	[75]
**P57**	2.07	−0.99/1.06	—	TEA	50.4^a^	5040^a^	2.31	[76]
**P63**	2.56	—	—	TEA	77.65^a^	3106^a^	13.2	[77]
**P64**	2.37	−1.02/1.35	Pt	TEOA	284.85^a^	5697^a^	6.1	[78]
**P65**	2.99	−1.86/1.15	—	TEA	147.1^a^	5884^a^	—	[79]
**P64**	2.31	−1.06/1.80	—	TEOA	400^a^	8000^a^	8.5	[80]
**P66**	1.95	−0.85/1.10	—	TEOA	366^a^	12200^a^	3.7	[81]
**P67**	1.85	—	Pt	AA	252.5^a^	10100^a^	0.6	[83]
**P68**	2.88	−1.79/1.09	—	TEA	30.66^b^	6132^b^	14.92	[84]
**P71**	2.12	−0.62/1.52	Pt	TEOA	45.4^a^	910^a^	2	[88]
**P74**	2.03	−0.69/1.34	Pt	TEOA	106^a^	2120^a^	0.48	[89]

^a^Light source: λ > 420 nm; ^b^light source: λ > 400 nm; ^c^light source: full-arc irradiation; ^d^light source: λ > 300 nm.

## Conclusion

Donor–acceptor interactions facilitate the ICT effect and decrease the optical bandgaps of D–A polymers. Correspondingly, they improve both charge carrier mobility and light harvesting, which makes D–A polymers potential photocatalysts for hydrogen production from water. In this minireview article, we systematically summarized the recent developments of the emerging diverse D–A polymers as photocatalysts for hydrogen evolution by water splitting. Several key messages could be concluded: (1) Architectures with multiple kind of acceptors, for example, D–A–A can improve the HER compared with simple D–A structures. (2) Incorporating heteroatoms, such as N, S, and O, into the polymer backbone can effectively modulate the physicochemical properties of the polymers. (3) Attaching side chains or functional groups, such as FSO and cyano groups, not only improve the solubility of CPs in water, but also tune the photoelectric properties of the polymers. Although the properties of CPs can be easily mediated by choosing different D and A building blocks, a good counterbalance between narrow bandgap and enough thermodynamic driving force for proton reduction is still hard to achieve for a single CP component.

In this respect, the combination of CPs with other semiconductors to form heterojunctions can further enhance the catalytic performance. Fabricating inorganic/polymer composites with complementary absorption ranges is an efficient strategy to extend the light-responsive region and offer enough thermodynamic driving force compared to single semiconductors. Besides, molecular heterostructures produced by two covalently bonded polymers could be a promising strategy because of effective charge carrier separation and small interfacial charge transfer resistance [103].

Consequently, we envisage the diverse kinds of CPs, in conjunction with inorganic semiconductors, promise enormous potential in PHP applications in the near future. Nevertheless, there is still a large potential for further improving D–A-type CPs for PHP applications. Large-scale and low-cost production of D–A CPs still remains a substantial challenge. Seeking atom- and step-economic synthetic alternatives [104] to traditional C-M/C-Br (M = B or Sn) coupling for preparing CPs ought to be a common goal. Furthermore, besides the PHP activity, studies regarding the long-term stability of CPs are still needed.
